# Generic drug policy in Australia: a community pharmacy perspective

**DOI:** 10.1186/1743-8462-4-7

**Published:** 2007-06-01

**Authors:** Grahame Beecroft

**Affiliations:** 1 Community pharmacist, Melbourne, Australia, Member of the Pharmacy Board of Victoria, Australia, Fellow of the Australian Institute of Pharmacy Practice and Management, Australia

## Abstract

This article provides a commentary, from a community pharmacy perspective, on the policy environment for the pharmacy sector in Australia, with a particular focus on present challenges arising from proposals to achieve substantial PBS cost savings from an anticipated surge of new generic drugs. Some $2 billion of medicines currently on the PBS will come off patent in the next 4 years. This growth comes from a low base where generics currently account for only 15% of the total PBS budget. Remuneration for PBS dispensing is fixed through five year agreements with the government, so trading terms on generics are important for the cross-subsidy of other dispensing activities and professional services. These trading terms (discounts provided by generics suppliers) have become part of the overall cost and revenue structure of pharmacies. Despite these arrangements, generic substitution rates in Australia are lower than in most comparable countries, which the government views as an opportunity to promote generic use. The future of generic drug supply via the PBS is important to allow consumers access to medications at the lowest possible price and to provide space for PBS listing of new and expensive drugs. But considerations of PBS reform need to take account of the role and viability of community pharmacy sector as provider of pharmaceuticals in a timely and efficient manner to Australian residents.

## Background

This article will address some of the issues currently facing community pharmacy, as a major wave of reforms to the Pharmaceutical Benefits Scheme (PBS) begins to take effect focusing on utilising an anticipated surge of new generic drugs to achieve substantial savings in the overall cost of the PBS. Community pharmacy comprises some 5 000 pharmacies throughout Australia which are part suppliers of Government funded pharmaceuticals and part retailers. This dual role is complex and unique, given also that community pharmacy provides much of the capital for the infrastructure that facilitates the delivery of pharmaceutical services to the Australian consumer. As a business entity, community pharmacy must ensure that it operates in a sound commercial environment, so any changes to the current financial parameters have major consequences. This article will explain some of the inter-related and inter-dependent relationships that make up community pharmacy, in the Australian setting, as the uptake of new generic drugs increases.

The definition of generic medicines accepted by pharmacists, suppliers to pharmacy and the professional organisations, is that generic drugs are originator or innovator products or products which are equivalent to originator brands, which are no longer protected by patent [1]. In this respect, a generic product range will include freshly off patent products (a current example would be pravastatin) and a large tail of different brands of older products (two examples would be amoxycillin and temazepam). However in the context of discussions of generics policy and PBS reform, the term generic typically excludes the off-patent original product and refers only to the alternative brand(s) marketed by other suppliers (or by the original supplier under a generic name).

An application for registration of a generic product generally includes a bioequivalence study which has the aims of establishing whether two or more formulations of the same drug are equivalent in terms of the rate and extent of absorption of the drug (or active moiety) into the systemic circulation.

In 1994, changes were made to legislation to permit pharmacists to substitute generic products for original brand pharmaceuticals if they are listed on the Schedule of Pharmaceutical Benefits as being bioequivalent and able to be substituted, even when the prescription specifies a particular brand. Under the legislation, substitution must not occur if the prescriber has indicated that substitution is not permitted.

Brand substitution may only occur after consultation with the patient or carer. The patient's health should always be the pharmacist's prime consideration in any brand substitution decision. Decisions to substitute one brand for another should not place patients at risk and pharmacists should endeavour to be consistent in the selection of brands for patients on long term therapy, to avoid patient confusion.

Pharmacists should encourage or offer to assist patients to have their medication regularly reviewed to check for duplication of different brands of the same medicine.

Generic medicine substitution will grow significantly due to three factors:

• There is a significant number of medicines that will become off-patent in the next 4 years. Some $2 billion of medicines currently on the PBS will come off patent.

• Government cost containment strategies to manage the growth of the PBS. These programs have been developed around increased support of generic substitution.

• Increased use of medicines due to an ageing population. Concession card holders are the most sensitive to the price benefits of generic brands over the originator products.

Remuneration for pharmacy is fixed because the price to the consumer is determined by the PBS. Medicines were priced in 2006 with a mark up of only 10% and a professional fee of $5.15 and a mark up of 4% or less applies to items priced over $450 [2]. For pharmacy to remain viable trading terms are therefore important. The trading terms relate to efficient purchasing and loyalty. Trading terms on generic medicines are used to cross-subsidise the bulk of other dispensing activities and professional services offered by community pharmacies. These trading terms include a discount level offered by generics suppliers and structured for each pharmacy, based around normal business drivers. As prices to consumers are fixed, pharmacists offer additional and varied services to consumers, to differentiate their pharmacy offer. Over the past decade, these trading terms have become part of the overall financial structure of pharmacies.

Community pharmacy by definition is service and knowledge focused; it is mostly located in convenient locations and is faced with the rising costs that all businesses face and some that are industry specific. Prescription business is a core activity for virtually every pharmacy and accounts for around 70% of pharmacy turnover.

Recently, various reforms have been introduced, based on the increased use of the freshly off patent drugs such as sertraline and simvastatin. The focus is squarely on these new generic drugs coming off patent as they are high priced, generally popular with both physicians and patients because of their improved efficacy compared to older products, generally have a higher safety index, are often presented in a dose form that encourages patient compliance (e.g. once a day dosing instead of three times a day) and have an acceptable cost to benefit ratio. The older, unprotected generic drugs are comparatively cheaper and have a lesser impact on the total costs of the Pharmaceutical Benefits Scheme.

Government programs and initiatives to increase the dispensing of generic medicines generate tensions when the financial structure of community pharmacy is such that increased generics use results in declining remuneration. Community pharmacy will find some difficulty in adjusting to this new set of government cost containment pressures.

## Generic pharmaceutical suppliers

Australia has a relatively small but active competitive landscape for generic pharmaceuticals with five major suppliers to the PBS [3]. Whilst a growing market for generic sales, Australia represents a logistical issue for the world's biggest generic players. The relatively small size of the market and high cost of entry make Australia a difficult investment opportunity for multi-national generic companies that are structured for high volume markets. But there is genuine competition in the market place, with active sales forces encouraging generic supplier loyalty and encouraging efficient, timely stock purchasing, either directly from the supplier or through a short line or full line wholesaler.

The actual supply of generics is a critical issue. The cost of storage and distribution is a real cost and is part of the obligation of wholesalers to offer all PBS items to community pharmacy, in any part of Australia, within 24 hours. When a drug is supplied by a full line wholesaler (either Sigma, API or Symbion) the pharmacist can order and receive any quantity and that item will be delivered as part of their next (usually) daily order. If the pharmacist orders the drug from the generic supplier (direct supply) or a short line wholesaler (offering a limited range of stock, usually the top sellers) the order may be subject to a volume requirement, so that the delivery of the stock is cost effective.

Considerable effort is expended to entrench supplier loyalty as pharmacists are reluctant to change generic suppliers for short term gains. Despite the fact that the generic marketplace in Australia is dominated by two larger companies, Alphapharm and Sigma Pharmaceuticals, the smaller generic companies are vigorous and nimble competitors.

## Australian generics growth

The average price of a generic is just over $13 while the average price of a PBS item is just over $40. In Australia, the growth of generics will exceed the growth of the PBS but comes from a low base where generics currently account for only 15% of the total PBS budget. The PBS growth has now dampened and is predicted not to exceed the rate of the economy growth for the foreseeable future [4]. Although only accounting for 15% of PBS dollars one in four prescriptions presented to pharmacists in Australia were filled with generic medicines in 2004–05. This level of use will almost double in the next five years as 58 of the 100 top selling prescription medicines will come off patent and be substituted by generic branded drugs [3].

A significant market factor is the increasing dominance that the emerging generics will have over the total dollar value of the market. By December 2008, 10 of the top 20 drugs will be patent expired. These 10 drugs currently represent 20% of the total dispensed cost of the PBS, a total of $1.3 billion and account for 15% of all the PBS prescriptions. At present only 2 of these drugs are patent expired.

In 2009 over 200 generic drugs will account for a dispensed value of around $2 billion. The top 10 generics will be responsible for 40% of this value ($800 m) and the top 40 generics will make up 85% ($1.7 b) [3].

This represents a significant shift in the current structure of the generic market for manufacturers and pharmacists. The patent expiry of such high value drugs will drive increased competition over a small part of the pharmaceutical product range and also concentrate pharmacy generic activity and profitability. This situation will be manifested in a numerically small number of generic drugs, compared to both the total generic range and the total number of listed pharmaceuticals, with a high dollar value and comprising the significant part of the total cost of the PBS. A satisfactory level of profitability is crucial for the ongoing survival and development of a competitive generics market in Australia and most fundamentally, the profitability of the generics market in community pharmacy underpins the professional service levels and business viability of community pharmacy and hence the welfare of Australian consumers.

## Generic medicines and consumers

When a consumer is supplied a generic medicine, the price to the consumer is generally lower than the branded product. This is complicated to an extent if the original brand medicine does not have a brand price premium (a manufacturer surcharge). In this situation the consumer would pay the standard concession price or the standard general price i.e. the price to the consumer is the same whether a generic or an original brand is supplied. However, as many original brands do have a brand price premium the consequent savings on medicines are of increasing importance when the consumer is taking multiple medications, on a long term basis. As the pharmacist may also be enjoying better trading terms if a generic medicine is supplied, it could be assumed that the consumer would be encouraged to consider using a generic instead of a branded product. Despite the more favourable trading terms, generic substitution rates in Australia are lower than in most comparable countries. It is for this reason the government wants to promote generic use.

The pharmacist must spend time with the patient to explain bio equivalence, packaging differences (for example compliance style blister strips, carton packaging which may be significantly different) and ongoing vigilance to avoid medicine error. The pharmacist must also talk to the consumer about differing brand names and drug names so that the patient appreciates the continuity of their medicines. The three parties involved in the medicine supply process (doctor, pharmacist and patient) may all use different names for the medicine, which is confusing and potentially risky. All these factors will affect compliance to medicines and therefore will affect health outcomes.

Consumers should not be expected to frequently receive a different brand of the same medicine. Pharmacists consistently balance competitive offers from generic suppliers with an offer that is reassuring for their patients. Consequently, major regulatory changes by government may result in unstable market conditions, which can affect consumers significantly, as pharmacists deal with the uncertainty of product supply and product pricing.

Generic medicines are no different to branded products in that patients must be offered counselling every time a prescription is dispensed. It is critical that consumers receive counselling by the pharmacist with every new medication, every change in dose and on a regular basis for those permanently taking a range of medicines for chronic conditions. Consumer Medicine Information, which provides an overview of relevant information to encourage effective medication use by the consumer and also details side effects and contra indications, is also offered to patients. Consumer Medicine Information is designed with the consumer in mind and is quite different to product information which is often found inside pharmaceutical packaging, and is written within a technical and legislative framework.

## Generic medicines and government

Government, through the Pharmaceutical Benefits Scheme, is running a reform agenda to achieve cost savings and efficiencies in the supply of medicines. Control in growth of the PBS has been a high priority within Treasury and the Department of Health and Ageing.

Price-volume agreements provide a mechanism for managing potential growth in PBS expenditure. The agreements represent a 'risk sharing' arrangement whereby any usage greater than an agreed level is subsidised at a lower price. This agreement recognises the fixed costs involved in the development of the drug and adjusts reimbursement as volumes increase, with only the variable costs increasing.

It is worth noting that when the PBS was in rapid growth – 14 per cent in 1999–00 and 19 per cent in 2000–01, the main drivers of this growth were the introduction of new blockbuster drugs onto the PBS, notably Celebrex and Zyban – and their enthusiastic prescribing by doctors, often for conditions for which they were not intended (e.g. 'leakage'). Government policy measure – risk share and price volume agreements imposed by the PBAC and PBPA will ensure such cost blowouts will not occur again [5].

Reference pricing ensures that Government reimbursement is equivalent to the lowest priced drug within each therapeutic group, known as the benchmark price. Drugs of similar safety, efficacy and health outcomes are placed in the same therapeutic groups at the same price and as new drugs are generally only listed on an equivalence basis, the size of reference groups is growing. A drug may only receive a higher subsidised price if the drug can be demonstrated to have a clinical advantage over alternative drugs.

Commencing in August 2005, when generic drugs are listed, the benchmark price is reduced by a mandatory 12.5%. The price reduction affects all brands of the drug and all forms and strengths of the drug that are administered in the same way. It also affects all other drugs which are deemed to be therapeutically equivalent.

The possibility of introducing a tendering system for the supply of PBS generics has also been part of the debate [6]. A simplistic attraction of a tendering system is the achievement of a low price for a set time. Long term ramifications of a tendering system are however seriously problematic. Alternative suppliers may simply cease to operate in Australia, as suppliers cannot maintain a business presence bereft of business activity. A single supply source is potentially risky for consumers and the changeover of medicine supply to consumers would create many difficulties related to compliance. The removal of an active, competitive market place for generics, as exists currently, changes the business environment totally. Business environments work best when competition is unfettered (of course in the pharmaceutical market, overarching legislative and professional obligations must be adhered to).

In November 2006, the Pharmacy Guild of Australia accepted the Government's Pharmaceutical Benefits Scheme (PBS) Reform Proposals. The Government will introduce major changes to the PBS. Medicines on the PBS will be separated into two groups, each subject to different pricing arrangements. The first group encompasses medicines where there is only a single brand listed (referred to as F1). This group contains both *on patent *and *off patent *medicines that are not substitutable with other brands or medicines. There are no mandatory price reductions for these medicines and existing price linkages are retained within this group.

The second group includes products for which there are many brands listed and groups of medicines that are interchangeable between patients (referred to as F2). There is already the requirement for a 12.5 per cent price reduction when the first new brand of a medicine is listed on the PBS. From 1 August 2008 a further reduction in the prices of these medicines will be required:

- a price drop of 2 per cent a year for three years for medicines where price competition between brands is low; and

- A one-off price drop of 25 per cent for medicines where price competition between brands is high

For example, medicines such as simvastatin, omeprazole, ranitidine, amoxicillin and felodipine have been identified as multiple brand medicines where there is high competition. Around 100 molecules currently costing the PBS $2 billion a year will fall into the high competition group.

The Government will move to a system of price disclosure, where the price that the Government pays will reflect the actual price at which the medicine is being sold.

Reference pricing links the price of a medicine to the price of other medicines that provide a similar health outcome. It will continue on F1 medicines in reference price groups and for F2 medicines that are interchangeable between patients. If a price change occurs for one of these medicines, this will flow to the others. [7].

## Generic medicines and pharmacists

Research shows that almost all community pharmacy net profits and cash flow have been negatively affected in 2005 and 2006 by the triple impacts of zero or negative PBS script growth, rising overheads and extreme competition. Data collected by the Pharmacy Guild of Australia (Guild Digest) and data collected from specialist accounting firms with many pharmacy clients (Johnston Rorke) support this claim [8]. Community pharmacy is no different to most retail activities, in that consumers seek competitive pricing. In pharmacy, competitive pricing occurs in every retail category from health and beauty to over the counter medicines. Price competition between pharmacies is robust. This is occurring as a result of focused marketing efforts by the larger branded or franchised stores, between the more traditional community pharmacies that operate under a franchise brand and groups of independently owned pharmacies that undertake promotional activities as a collective. In much of the pharmacy market, there is also competitive activity with other retailers such as supermarkets. A point often missed is that community pharmacy provides an alternative in this retail segment to dominance by the supermarket sector; its presence provides some balance, although small, to the current retail duopoly.

Government initiatives such as the 20 day rule (for certain PBS medicines a repeat supply of the same medicine within 20 days will fall outside the Safety Net), increased patient co-payments (where the patient pays an increased proportion of the total cost of medicines supplied) and the increased safety net qualification have all slowed script volumes and sales. Pharmacists observe that many consumers do have difficulty in funding pharmaceutical purchases; this is particularly the case for consumers who do not qualify for a concession card and may be taking three to five items permanently. The monthly cost for these consumers is between $100 and $150. The Australian Pharmaceutical Benefits Scheme provides a significant subsidy over the total real cost for such pharmaceuticals, but nevertheless, pharmacists observe first hand the financial difficulty that many consumers experience. Of major concern is the unknown impact that failure to take medicines can have on both consumer wellbeing and the overall health budget. Non concession card consumers can obtain some relief if they qualify for a safety net card, although the dollar target for this still excludes many (in 2007 $ 1059 per annum). The recent 5-year Fourth Community Pharmacy Agreement was negotiated on the basis of pharmacist remuneration increasing by 30% over the 5 years. In the first year of the agreement the PBS grew by a mere 1.7%, while volume actually fell 1% in the same period. Based on the original estimates for the five years it is now expected the PBS under spend will be $2 billion. This $2 billion savings is based on the existing PBS model and does not take into consideration the new Abbott proposals.

Overheads, particularly pharmacist wages, have increased at double digit growth rates for the last 3 years due to the shortage of health professionals [8]. Research shows that most pharmacies are suffering a downturn in net profits. This is despite the increased availability of generic drugs and the generic supply rate increasing from 18% to 25% (and pharmacists securing better trading terms compared with branded products). The actual net profit seems to depend heavily on the improved trading terms. Ironically, in the future, pharmacies may find they receive a lower margin with generics than on branded medicines. This would be a major disincentive to encourage the use of generics.

The supermarket industry and discount department stores have been taking market share from pharmacy, selling non scheduled products, which places more importance on dispensing and OTC (Over The Counter) medicines.

## Viability of Community Pharmacy: impact of using generic drug policy to drive down PBS costs

Pharmacies are part-Government medicines dispenser and part-retailer. A typical pharmacy generates sales of around $2 million per year, and runs at a gross margin of 27%–32% of sales. The major costs for pharmacy are rent and wages. Rent is a significant issue because pharmacies are best located in busy retail locations, usually close to other everyday shopping activities (chore shopping). Consumers expect easy access to their pharmacy and this is essential as key pharmacy shoppers are the elderly and the less mobile.

Pharmacist wages are rising because there is an overall shortage of some 3000 pharmacists in the Australian market. Pharmacists are required across extended hours and during weekends and public holidays. Additionally, Australian Pharmacy Boards, which primarily represent the interests of consumers, mandate that pharmacists should practice in a safe manner. A safe work load is, according to most professional organisations, somewhere between 100 and 150 prescriptions per pharmacist during an 8 hour day. This assumes that the pharmacist in also performing a range of other tasks: communicating with doctors, supervising the sale of scheduled medicines, assisting consumers with a wide range of health problems, administering pharmacotherapy programs and supervising other staff. A pharmacy technician is also usually present to assist the pharmacist. Tasks that the technician may undertake are ordering and packing away stock, clerical duties, cleaning duties and customer service.

Pharmacies must also provide for other occupancy costs and maintain the pharmacy in a professional and efficient manner. Pharmacies should also allocate funds for timely physical improvements, both ongoing and for major periodic refurbishments. Consumers expect a pharmacy to be modern, bright and efficient and with enough space to shop comfortably and importantly to receive confidential counselling when required. Current refurbishments cost around $1,000 to $ 2,000 per square metre and with community pharmacies ranging in size from 250 to 400 square metres, refurbishment costs are significant. Also, many smaller pharmacies are seeking to increase their current footprint and to introduce efficiencies, but expansion requires access to funding and growth opportunities.

Individual pharmacists invest in pharmacy businesses for two reasons. Firstly, as a health practitioner, they feel that even within overarching legislative and professional jurisdictions, there is still an opportunity to practice their profession in their preferred style and in effect, make their own unique offer to consumers. This individuality of offer is positive for consumers, in that there is an opportunity to seek out the pharmacist that best suits their needs. Secondly, pharmacists invest in pharmacy businesses to earn a return on their investment and to maximise that return by investing management skills into the business. However, given that 70% of business activity is PBS prescriptions and that prescription prices to the consumer are fixed and also that mark up and wholesale prices are fixed, the pharmacist has little control in driving profitability of the PBS component of the business.

This is a crucial issue for community pharmacy owners as they are also facing reduced margins due to current PBS initiatives, particularly measures that involve the use of generic medicines, in a climate of almost zero growth. Pharmacists have virtually no control over the PBS drivers. Gross Profit has been further eroded by the impact of the low 10% mark up on expensive drugs and the flat mark up of $40 for drugs priced over $1,000. Net profit has been largely positively influenced by trading terms, both generally and via generic suppliers. As any business owner will attest, improvements in trading terms are hard earned – they are usually a reward for loyalty and efficient purchasing practices.

Pharmacists have traditionally offered consumers a range of services which complements the core activity of dispensing. Consumers have enjoyed low cost or free filling of Dose Administration Aids, free delivery to the less mobile, support services for nursing homes and significant free advice to consumers, often without the sale of a product. These services have been provided out of the 'general profitability' of funds available to the pharmacy business owner.

Further reduction in margin on prescriptions would render many of these services unviable. In order for pharmacies to maintain liquidity, major changes to the cost structure of pharmacies would need to be implemented. The first option would be to reduce the number of pharmacists in pharmacy, thereby changing the practice of pharmacy as we know it. Pharmacies would become 'sausage factories' churning out prescriptions, with very little time to counsel patients. The second option may be to introduce fees for services currently provided at no cost. When price is the only determinant, does the consumer receive the best service and the best outcome? Observations in other countries indicate a paucity of service levels.

Pharmacists may also look to expand their retail sales, in effect using the PBS traffic to create profitable non-prescription sales.

It will not be possible for a retail solution to save pharmacies, so that they may continue to supply PBS prescriptions on behalf of the government. It is probable that many pharmacies would suffer severe financial hardship forcing closure, or reach such a low state of profitability that they may be unable to provide a wide range of services. Some pharmacies may find that their way forward would be to focus almost solely on retail sales, with very little or no advice available on prescriptions.

## Concluding discussion

Although the PBS is in decline in terms of prescriptions dispensed and PBS expenditure is growing more slowly than the economy, the government continues to reform the PBS, via a series of initiatives, mostly centering on changes to generic medicines. New drugs are only added to the PBS if they satisfy a rigorous cost benefit analysis, and they are most often priced at the therapeutic group bench mark price. The imposition of mandatory price cuts each time a generic is listed on the PBS may well start to lessen the interest of generic companies in listing their products.

The financial effect on community pharmacy will not be known for some time but the recent reforms will deliver new financial dynamics into every pharmacy business.

Community pharmacy, because of its core activity of supplying PBS prescriptions, is already showing nil or negative growth. Mandatory counselling services provided by pharmacy may be lost if pharmacy owners need to reduce the number of pharmacists employed. Consumers have a right to enjoy contemporary pharmacy services in Australia and these services cannot happen unless a satisfactory financial business model is in place.

The future of generic drug supply via the PBS is critically important to ensure a cost effective and competitive drug supply pipeline for Australians, to allow for new and expensive drugs to be listed on the PBS when appropriate and to allow consumers access to medications at the lowest possible price. The current PBS reforms will deliver price transparency for each generic supplied to pharmacy and the true cost price of each drug will be known to all parties. Historically, the discounts given by generic manufacturers to pharmacists have become part of the overall profit mix of the pharmacy. New incentive payments have been introduced so that pharmacists will be able to replace some part of the forgone generic discounts, and it is hoped that this new overall profit figure will continue to underpin the services that pharmacy offers to consumers. This subtle shift of identifying the lowest actual cost of a drug, with full transparency, may lead to clearer recognition of the range of professional services that a pharmacist provides in every patient/pharmacist interaction. The cost of such professional services may be debated more fully in the future and the quantification of these services will recognise the finite capacity of the pharmacist and the real costings in the Australian setting.

Community pharmacy is unsure whether the new incentive payments will be an adequate compensation, however it recognises that generic price transparency will be a standard practice and that generic manufacturers, perhaps the most directly affected by the new reforms, will offer significantly different trading terms to the trading terms experienced more recently.

## Competing interests

The author has a proprietary interest in three community pharmacies.
